# An MXene nanocomposite hydrogel for enhanced diabetic infected wound healing via photothermal antibacterial properties and bioactive molecule integration

**DOI:** 10.1016/j.mtbio.2025.101538

**Published:** 2025-02-08

**Authors:** Xue Ou, Zhijie Yu, Xi Zheng, Le Chen, Chuanyu Pan, Dandan Li, Zhenzhen Qiao, Xiaoyuan Zheng

**Affiliations:** Pharmacy Department, Chongqing Emergency Medical Center, Chongqing University Central Hospital, Medical College, Chongqing University, Chongqing, 400014, China

**Keywords:** Diabetic wounds, Ti₃C₂Tx-Ag, Nanocomposite hydrogel, Photothermal antibacterial, Wound healing

## Abstract

Diabetic wounds are a major clinical challenge due to their chronic, non-healing nature, which significantly impacts patients' quality of life. Traditional treatments often fail to effectively promote wound healing, highlighting the need for new biomaterials. In this study, we developed a composite hydrogel (KC@PF@TA) that combines the photothermal and antibacterial properties of Ti₃C₂Tx-Ag (Titanium carbide-silver) with the regenerative effects of paeoniflorin (PF). The hydrogel was optimized by adjusting the composition, crosslinking density, and the incorporation of nanoparticles, which enhanced its mechanical strength, photothermal conversion efficiency, antibacterial properties, and biocompatibility. The optimized hydrogel demonstrated enhanced cell proliferation, migration, and robust photothermal and antibacterial properties in vitro. In a diabetic murine model of *Staphylococcus aureus*-infected wounds, KC@PF@TA exhibited exceptional therapeutic benefits in antibacterial, anti-inflammatory, angiogenic, and tissue regeneration. Overall, our results suggest that composite hydrogels with controlled bioactive agent release and mechanical modulation present a promising solution for treating chronic diabetic wounds.

## Graphical abstract

**Figure:**
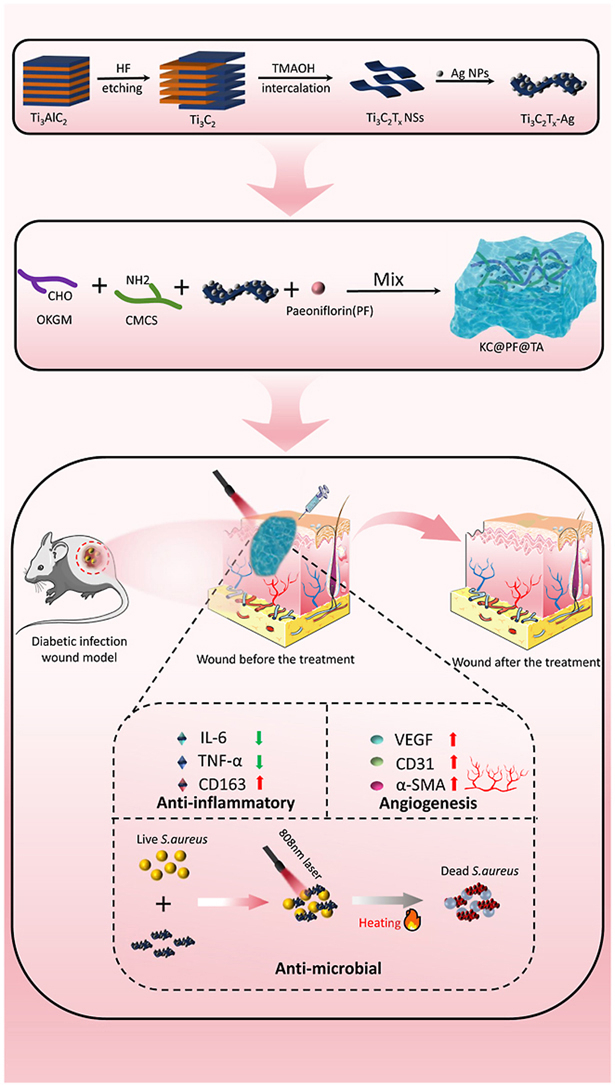

## 1 Introduction

Diabetes mellitus is a chronic metabolic disorder marked by persistent hyperglycemia, leading to various complications, including impaired wound healing [1,2]. Chronic wounds, particularly diabetic ulcers, present a significant clinical challenge due to their slow healing, high risk of infection, and frequent recurrence [3,4]. Approximately 15 % of diabetic patients worldwide will develop at least one stubborn foot ulcer during their lifetime, with around 20 % of these cases ultimately resulting in lower limb amputation [5,6]. Moreover, diabetic patients face a four-fold increased risk of wound infection compared to non-diabetic individuals, making it a global health concern [7]. The poor healing of diabetic wounds is primarily due to angiogenesis impairment, chronic inflammation, and excessive oxidative stress [8,9]. Although treatments such as negative pressure therapy, skin substitutes, and growth factor therapies are available, their effectiveness in addressing chronic inflammation and promoting angiogenesis remains limited, failing to fully meet clinical needs. Consequently, there is a pressing need for innovative therapeutic materials that can target these challenges, accelerate healing, and reduce complications.

Recent advancements in biomedical materials have led to the development of multifunctional hydrogels designed to improve wound healing [10]. These hydrogels, typically composed of biocompatible and biodegradable polymers, possess high water retention, creating a moist environment that promotes cell migration and tissue regeneration [[11], [12], [13]]. Additionally, they can serve as drug delivery systems, offering controlled release of antibiotics, anti-inflammatory agents, or growth factors directly to the wound site, thereby enhancing drug concentration and bioavailability to accelerate healing [14,15]. However, the practical application of hydrogels continues to face several challenges. Traditional hydrogels often suffer from low mechanical strength, making them susceptible to rapid degradation in bodily fluids [16,17]. This limitation reduces their capacity to sustain drug delivery and maintain coverage over large or deep wounds for extended periods. Additionally, controlling the drug release rate remains problematic, frequently leading to an initial burst of excessive drug release followed by inadequate drug availability in later stages [18].

Nanocomposite hydrogels have garnered significant attention due to their ability to combine the structural benefits of hydrogels with the functional advantages of nanomaterials [19,20]. This synergy allows the creation of materials that not only support wound structure but also enhance drug delivery and actively regulate the local microenvironment to aid in the healing process [[21], [22], [23]]. Various nanomaterials, including copper, zinc, silver, graphene oxide, and MXenes, have been incorporated into hydrogels to improve their physicochemical properties [24]. Ti₃C₂Tx, a type of MXene, stands out among other MXenes due to its excellent biocompatibility, robust mechanical properties, and significant potential for biomedical applications, particularly in wound healing [25]. Its unique combination of properties, including strong photothermal conversion efficiency, plays a critical role in enhancing the antibacterial properties of hydrogels when exposed to near-infrared (NIR) light [26]. The ability of Ti₃C₂Tx to generate reactive oxygen species (ROS) further strengthens its antibacterial efficacy, making it particularly effective in combating bacterial infections, a key concern in wound care [27]. Additionally, Ti₃C₂Tx demonstrates superior dispersibility in aqueous solutions, which is essential for its uniform integration into hydrogel matrices [[28], [29], [30]]. Its large surface area provides numerous active sites for the adsorption and controlled release of therapeutic agents such as antibiotics and anti-inflammatory drugs, which is crucial for the long-term management of diabetic wounds where sustained therapeutic effects are required [[31], [32], [33], [34], [35]].

Despite the excellent properties of Ti₃C₂Tx-based hydrogels, their inability to regulate complex biological processes, such as inflammation resolution, angiogenesis, and tissue regeneration, limits their broader application in wound healing. To address these challenges, the integration of traditional Chinese medicine components into hydrogels provides a multifunctional synergistic strategy to enhance wound healing outcomes [36,37]. PF, a bioactive monoterpene glycoside extracted from *Paeonia lactiflora*, has been extensively studied for its anti-inflammatory, antioxidant, and pro-angiogenic properties [38,39]. PF effectively reduces the expression of pro-inflammatory cytokines, promotes macrophage polarization toward the M2 phenotype, and enhances the migration and proliferation of endothelial cells, thereby facilitating angiogenesis and accelerating tissue repair [40]. These advantages position PF as a promising candidate for overcoming the limitations of Ti₃C₂Tx hydrogels. Recent studies have demonstrated PF's efficacy in various wound-healing applications, particularly in diabetic wounds, due to its ability to modulate the wound microenvironment and promote regenerative processes [41,42].

**Scheme 1:**
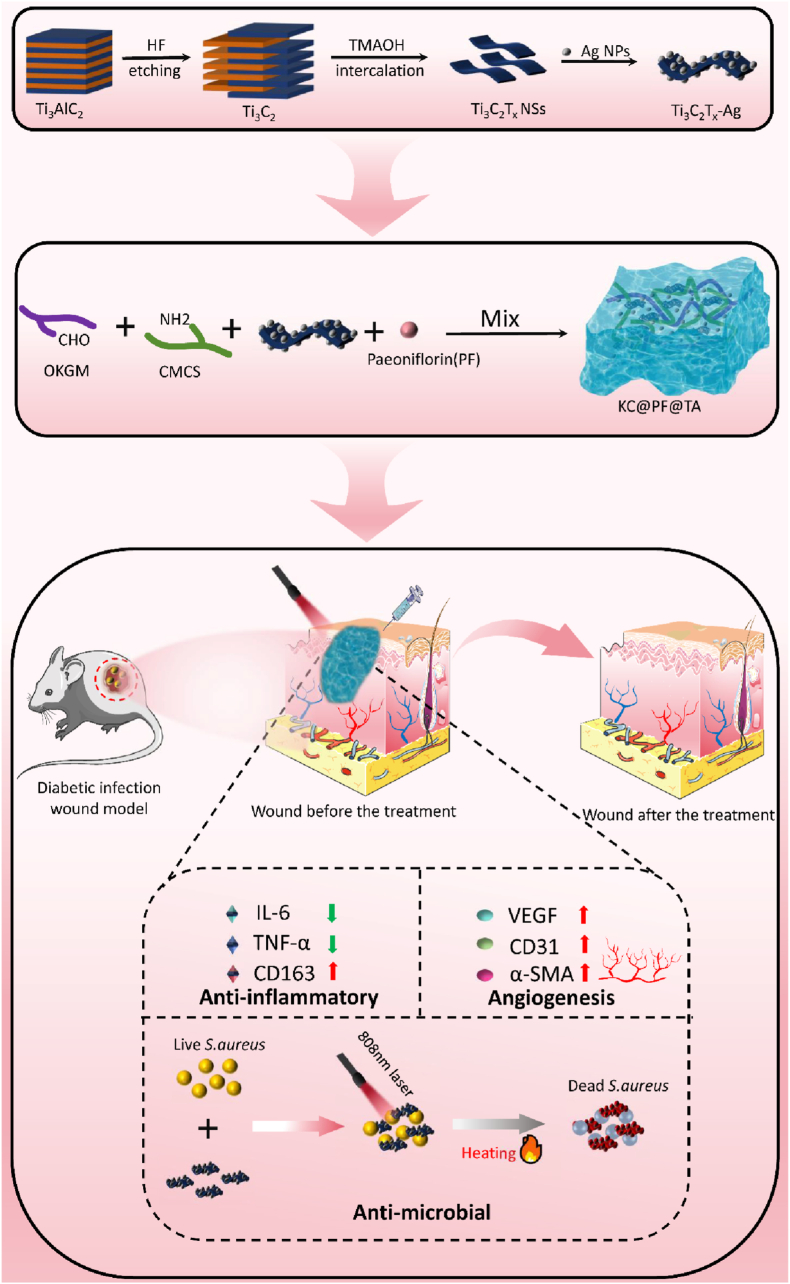
Schematic representation of the preparation process of Ti_3_C_2_T_x_-Ag nanocomposite, KC@PF@TA hydrogel and the treatment of diabetic infected wounds. The KC@PF@TA hydrogel enhanced diabetic wound healing by anti-inflammation, promoting angiogenesis, and antibacterial resulting in skin tissue regeneration with mature epithelial structures.

In this study, we designed a nanocomposite hydrogel (KC@PF@TA) to leverage the photothermal antibacterial properties of Ti_3_C_2_T_x_ enhanced by silver (Ag) nanoparticles and the anti-inflammatory, angiogenic, and cell proliferation-promoting effects of PF. The hydrogel demonstrated excellent biocompatibility, injectability, and mechanical properties, making it suitable for treating irregular wounds. The KC (oxidized konjac glucomannan and carboxymethyl chitosan) component of the hydrogel facilitated sustained drug release, which in turn reduced inflammation, enhanced antibacterial effects, and promoted cell proliferation and angiogenesis, continually driving wound revascularization. To validate our approach, we investigated the interactions between Ti_3_C_2_T_x_ and Ag, evaluated the hydrogel's properties, and assessed its antimicrobial efficiency, cell viability, and biocompatibility in vitro. Finally, the hydrogel was applied to treat full-thickness infected wounds in a diabetic model. The results showed that the KC@PF@TA hydrogel accelerated wound closure by inhibiting inflammation, promoting angiogenesis and M2 macrophage expression, and enhancing re-epithelialization, collagen deposition, and neovascularization, providing a comprehensive solution for diabetic wound treatment (Scheme 1).

## 2 Materials and methods

### 2.1 Materials

The Ti₃AlC₂ powder (98 %), hydrofluoric acid (HF, CP, 40 %), tetramethylammonium hydroxide (TMAOH, AR, 25 %), silver nitrate concentrate (AgNO_3_), ethylene glycol (AR, 98 %) were purchased from Aladdin (Shanghai, China). Anhydrous sodium citrate (SC) was obtained from Thermo scientific (USA). Carboxymethyl chitosan (Carboxymethylation≥80 %, 100–200 kDa), konjac glucomannan (purity≥95 %, 500-2000 kDa) and sodium periodate (99.5 %) were purchased from Macklin (Shanghai, China). Dialysis membreanes (12,000–14,000 kDa) was obtained from Yuanye (Shanghai, China). The LB broth (BR), Agar powder and Calcein-AM/PI staining kit were obtained from Solarbio (Beijing, China). RIPA lysis buffer and PSMF were purchased from Beyotime (Shanghai, China). ELISA kits for tumor necrosis factor-α(TNF-α), interleukin-6 (IL-6), and vascular endothelial growth factor (VEGF) were purchased from Meilian (Shanghai, China). *Staphylococcus aureus* (ATCC 25923) was obtained from Ningbo testbio Co., Ltd (Zhejiang, China).

### 2.2 Synthesis of Ti₃C₂Tₓ nanosheets

The 2 g of Ti₃AlC₂ powder were gradually added to 20 mL of 40 % HF solution over 5–10 min, followed by stirring at 550 rpm for 24 h at 35 °C. The resultant mixture was then centrifuged at 3500 rpm for 15 min. The supernatant was discarded, and the precipitate was washed 5–7 times with 15 mL of deionized water. Washing continued until the pH of the supernatant was approximately neutral, as confirmed by pH test paper. Subsequently, 25 % TMAOH was added to the precipitate, and the mixture was stirred at 550 rpm for 24 h at 35 °C. After centrifugation, the precipitate was again washed with deionized water until neutral pH was reached. The final precipitate was resuspended in deionized water and sonicated for 2 h. The suspension was then centrifuged at 3500 rpm for 1 h. The resulting upper colloidal solution was collected and subjected to vacuum freeze-drying for 24 h, yielding u-Ti₃C₂Tₓ nanosheet powder.

### 2.3 Preparation of the u-Ti₃C₂Tₓ-Ag composite

A 30 mg quantity of u-Ti₃C₂Tₓ was dispersed in 30 mL of deionized water and subjected to sonication for 30 min to achieve uniform dispersion. The resulting mixture, referred to as Solution A, was stirred for 5 min at room temperature. Solution A was then slowly added to a 5 mM AgNO₃ solution at u-Ti₃C₂Tₓ to Ag mass ratios of 1:1, 1:5, and 1:10. The mixture was stirred for 1 min before being transferred to a microwave oven. The solution was heated to boiling, after which a predetermined amount of 1 % SC was added. The mixture was then heated for an additional 30 min. After cooling to room temperature, the resultant powder was centrifuged at 13,000 rpm for 15 min, washed six times with deionized water, and vacuum freeze-dried for 48 h to yield the u-Ti₃C₂Tₓ-Ag powder, hereafter referred to as Ti₃C₂Tₓ-Ag.

### 2.4 Preparation of the nanocomposite hydrogel

Oxidized Konjac glucomannan (OKGM) and Carboxymethyl chitosan (CMCS) were selected as hydrogel matrix materials based on previous studies [43]. A 10 mg quantity of Ti₃C₂Tₓ-Ag powder was ultrasonically dispersed in deionized water to obtain a 1 mg/mL dispersion. CMCS powder was then added and fully dissolved to create Solution A, with a mass fraction of 3 % CMCS. In parallel, OKGM was fully dissolved in deionized water to produce Solution B, with a mass fraction of 6 %. Equal volumes of Solutions A and B were combined in a Teflon mold and mixed thoroughly. The resulting KC@Ti₃C₂Tₓ-Ag hydrogel (hereafter referred to as KC@TA) was then vacuum freeze-dried to obtain the final sample.

### 2.5 Characterization of composite and hydrogel

Environmental scanning electron microscopy (SEM, Quattro S, ThermoFisher Scientific, DE, USA), transmission electron microscopy (TEM, Talos F200S, ThermoFisher Scientific, DE, USA), X-ray diffraction (XRD, PANalytical X'Pert Powder, LON, UK) and X-ray photoelectron spectroscopy (XPS, ESCALAB250Xi, ThermoFisher Scientific, DE, USA), Fourier transform infrared spectroscopy (FTIR, Nicolet iS50, ThermoFisher Scientific, DE, USA) were used to characterize the morphology and physicochemical properties of all samples.

### 2.6 Comprehensive performance testing of hydrogels

Self-healing and Injectable performance: A piece of hydrogel was cut into two separate pieces. These pieces were then rejoined to form a single, continuous piece, and the sample was left at 25 °C for 10 min to observe the self-healing capability. Additionally, the hydrogel was loaded into a 1 mL syringe and gently pushed to assess its injectability and the smoothness of the extrusion.

Swelling: The dried KC@TA hydrogel was immersed in 500 mL of deionized water at room temperature. At specific time intervals (0, 1, 2, 3, 4, 5, 6, 7, 8, 12, and 24 h), the hydrogel was carefully removed, and excess surface water was gently blotted using dry filter paper. The weight of the hydrogel was recorded at each time point. The swelling ratio was calculated using the following equations:

(1-1) $$\text{Swelling}\;\text{ratio}\%=\left(W_{t}-W_{0}\right)/W_{0}\times 100\%$$

where W_0_ and W_t_ represent the initial mass, the mass at different time of the hydrogel, respectively.

Degradation: To assess the degradation behavior, the hydrogel was first allowed to reach swelling equilibrium and then weighed to obtain the initial mass W_i_. The hydrogel was subsequently immersed in 100 mL of normal saline (0.9 % NaCl). At daily intervals (from day 0 to day 12), the hydrogel was removed, blotted dry with absorbent paper, and weighed as W_f_. The percentage weight remain of the hydrogel was calculated using the following equation:

(1−2) $$\text{Remaining}\;\text{weight}\%=W_{f}/W_{i}\times 100\%$$

where W_i_ and W_f_ represent the initial mass and the mass of the hydrogel at each time point, respectively.

### 2.7 Measurements of photothermal performance

Photothermal Heating: A series of aqueous solutions of Ti₃C₂Tₓ and Ti₃C₂Tₓ-Ag were prepared at concentrations of 0, 25, 50, 100, and 200 μg/mL. Each solution (100 μL) was added into separate wells of a 96-well plate. The plate was covered with aluminum foil and equilibrated at room temperature for 10 min to ensure uniform initial temperatures. Subsequently, each sample was irradiated with an 808 nm NIR laser at a power density of 1.2 W/cm^2^ for 5 min. Temperature changes were recorded every 30 s using an infrared thermal imaging camera.

Photothermal Stability: A 100 μL aliquot of Ti₃C₂Tₓ-Ag solution at a concentration of 200 μg/mL was placed into a well of a 96-well plate. The sample was subjected to cyclic irradiation using an 808 nm NIR laser at a power density of 1.2 W/cm^2^ for 3 min, followed by natural cooling to room temperature for another 3 min. This cycle was repeated five times. Throughout the process, temperature measurements were taken at 20-s intervals using an infrared thermal imaging camera to assess the photothermal stability of the Ti₃C₂Tₓ-Ag solution.

Comparative Photothermal Analysis*:* TA and KC@TA hydrogel samples were prepared at a concentration of 200 μg/mL, with each sample volume set to 400 μL and injected into separate Eppendorf tubes. The samples were irradiated with an 808 nm NIR laser at a power density of 1.2 W/cm^2^ until the temperature plateaued, indicating a steady-state condition. After irradiation, the samples were allowed to naturally cool to room temperature. Temperature readings were captured every 30 s during the irradiation period using a thermal imaging camera to compare the photothermal performance between Ti₃C₂Tₓ-Ag and KC@TA hydrogels.

### 2.8 Ag^+^ releasing assay

The concentration of Ag^+^ in the KC hydrogel was determined using inductively coupled plasma mass spectrometry (ICP-MS, NexION 5000, Perkinelmer, MA, USA). Briefly, hydrogels were prepared according to previous protocols. Ti₃C₂Tₓ and TA hydrogels, with a concentration of 1 mg/mL, were immersed in 10 mL of deionized water at 37 °C. The samples were subjected to 808 nm NIR irradiation at a dose of 1.2 W/cm^2^ for 10 min every 24 h. After each irradiation, 200 μL of the solution was extracted and replaced with 200 μL of fresh deionized water. The Ag^+^ concentration in all samples was measured by ICP-MS, with sampling points taken from day 0 to day 6.

### 2.9 In vitro antibacterial activity assay

The antibacterial activity of hydrogels against *Staphylococcus aureus* (ATCC 25923) was investigated in vitro using plate colony counting and SEM was used to observe the bacterial morphology. Equal volumes of KC, KC@T_200_, and KC@TA_25/50/100/200_ μg/mL) hydrogels were sterilized by immersion in 75 % ethanol for 2 h. Following sterilization, the hydrogels were rinsed three times with sterile PBS to remove residual ethanol and then placed in a 24-well plate. A 10 μL aliquot of bacterial suspension (10⁶ CFU/mL) was applied to the surface of each hydrogel sample. The bacteria were incubated on the hydrogel surface for 15 min, with or without exposure to 808 nm NIR laser irradiation at a power density of 1.2 W/cm^2^. Bacteria incubated in blank wells, with or without NIR irradiation, served as the control group. After treatment, 990 μL of sterile PBS was added to each well to resuspend the surviving bacteria. A 10 μL aliquot of the resuspended bacteria was then plated on Agar and incubated at 37 °C in a constant temperature incubator. After 18–24 h, colonies on the Agar plates were counted. The bacterial survival rate was calculated by comparing the number of colonies in each experimental group to the control group, where the colony count without NIR irradiation was set as the reference value.

The remaining bacterial suspension was centrifuged at 3000 rpm for 5 min, the supernatant was discarded, and the bacterial pellet was fixed and dehydrated as follows. First, 1 mL of sterile PBS was added to the bacterial pellet, gently resuspended, and then centrifuged again at 3000 rpm for 5 min. This washing step was repeated three times. After washing, 1 mL of 4 % glutaraldehyde was slowly added to the pellet, mixed thoroughly, and incubated at 4 °C for 4 h to fix the bacteria. The suspension was then centrifuged at 3000 rpm for 5 min, the glutaraldehyde was discarded, and the pellet was washed three times with 1 mL of PBS, followed by centrifugation to collect the bacterial pellet. Next, 2.5 % glutaraldehyde was added, and the mixture was left to settle at 4 °C for 1 h to obtain the fixed bacterial pellet. This pellet was washed three times with 1 mL of PBS and centrifuged each time to ensure thorough washing. For dehydration, 1 mL of 20 % (w/v) ethanol was added to the pellet, gently mixed, and left for 10 min before centrifuging. This was followed by sequential dehydration using 1 mL of 50 %, 80 %, and 100 % (w/v) ethanol, each for 10 min with subsequent centrifugation. Finally, the remaining ethanol was removed by gentle swinging. A small amount of the dehydrated bacterial sample was then transferred using a toothpick onto conductive adhesive. The sample was sputter-coated with gold, and the bacterial morphology was observed using a scanning electron microscope.

### 2.10 Cell proliferation and migration assay

The CCK-8 assay was employed to assess the proliferation of human umbilical vein endothelial cells (HUVECs). Cells were seeded into 96-well plates at a density of 5 × 10³ cells per well, with each well containing 100 μL of medium. After 24 h, the cells were treated with PF at concentrations of 0, 3.125, 6.25, 12.5, 25, 50, 100, and 200 μM. Following 48 h of incubation, the medium was removed, and 100 μL of fresh medium containing 10 % CCK-8 solution was added to each well. The plates were then incubated at 37 °C in a 5 % CO₂ atmosphere for 1.5 h. Optical density (OD) at 450 nm was measured using an inverted fluorescent microscope (Synergy H1, BioTek, VT, USA). The cell proliferation rate was calculated using the following formula:

(1–3) $$\text{Proliferation}\;\text{ratio}\%=\left[\left({\text{OD}}_{s}-{\text{OD}}_{b}\right)/\left({\text{OD}}_{n}-{\text{OD}}_{b}\right)\right]\times 100\%$$

where OD_b_, OD_n_, and OD_s_ represent the OD450 for the blank group, negative control group, and experimental group, respectively.

The cell migration ability was evaluated using a scratch assay. HUVECs were seeded into 6-well plates at a density of 1 × 10⁶ cells per well and cultured until they reached full confluence. A 200 μL pipette tip was used to create three parallel scratches in each well. Subsequently, each group was treated with 2 mL of 10 % DMEM containing PF at concentrations of 6.25, 12.5, and 25 μM. The cells were incubated at 37 °C in a 5 % CO₂ atmosphere. The scratch width was observed under bright-field microscopy using an inverted fluorescent microscope (IX73, Olympus, Japan) and quantified using ImageJ software.

### 2.11 Biocompatibility examination

PF was incorporated into the hydrogel matrix to create the KC@PF@TA hydrogel, and its hemolytic activity was evaluated. According to the national standard GB/T16886.5, 1 g of KC@PF@TA hydrogel was sterilized under ultraviolet light for 2 h and immersed in 10 mL of complete medium, followed by extraction in a 37 °C water bath for 24 h. After filtration through a 0.22 μm membrane to remove bacteria, the extract was sealed and stored at 4 °C. NIH3T3 cells in the logarithmic growth phase (5 × 10^3^) were seeded into 96-well plates with 100 μL of cell suspension per well. Following overnight incubation to allow cell adherence, the old medium was replaced with 200 μL of either fresh complete medium or KC@TA_25/50/100/200_ hydrogel extract. Each group included five replicates. After adding the extract, the cells were incubated at 37 °C in a 5 % CO₂ atmosphere. At 24 h and 48 h post-treatment, the medium was removed, and 100 μL of 10 % CCK-8 solution was added to each well, followed by incubation in the dark for 1–2 h. Absorbance at OD450 was measured using an inverted fluorescent microscope. The cytotoxicity was calculated using the following formula for in vitro cell viability:

(1–4) $$\text{Cell}\;\text{viability}\%=\left[\left({\text{OD}}_{s}-{\text{OD}}_{b}\right)/\left({\text{OD}}_{n}-{\text{OD}}_{b}\right)\right]\times 100\%$$

where OD_b_, OD_n_, and OD_s_ represent the OD450 for the blank group, negative control group, and experimental group, respectively.

Additionally, cells from different treatment groups cultured for 48 h were stained with a Calcein-AM/PI staining kit to observe the growth and proliferation of NIH3T3 fibroblasts.

### 2.12 Hemolytic test

Blood was collected from the mice via orbital puncture into anticoagulant tubes, followed by centrifugation at 3000 rpm for 15 min. The supernatant was discarded, and the red blood cells were resuspended in normal saline and centrifuged again. Normal saline served as the negative control, and deionized water was used as the positive control. The KC and KC@PF@TA samples were immersed in an equal volume of deionized water, and then 250 μL of 5 % red blood cell suspension was added. The mixtures were incubated in a 37 °C water bath for 30 min, followed by an additional hour of incubation to observe any signs of hemolysis. After incubation, the mixtures were centrifuged at 3000 rpm for 5 min, and 200 μL of the supernatant was transferred to a 96-well plate. The OD540 was measured using an inverted fluorescent microscope, with three parallel samples per group. The hemolysis rate was calculated using the following formula:

(1–5) $$\text{Hemolysis}\;\text{rate}\%=\left({\text{OD}}_{s}-{\text{OD}}_{n}\right)/\left({\text{OD}}_{p}-{\text{OD}}_{n}\right)\times 100\%$$

where OD_s_, OD_n_, and OD_p_ represent the OD values of the experimental group, negative control, and positive control, respectively.

### 2.13 In vivo wound healing experiments

5-week-old C57BL/6J male mices were selected and fed a high-fat diet (60 % kcal) after one week of adaptive feeding. After 4 weeks of high-fat diet, STZ mice were intraperitoneally injected with streptozotocin (pH = 4.2–4.5 citrate buffer) at 30 mg/kg [44]. Before the intraperitoneal injection, the mice were fasted for 12 h overnight, and the injections were continued for 5 days, with food provision resumed 2 h after each injection. Random blood glucose was measured and recorded after 3–5 days. Mice with blood glucose levels >16.7 mM were considered to have successfully established type 2 diabetes.

The mice were anesthetized with an anesthesia machine, and the back was shaved. The fascial layer was cut out with a diameter of 6 mm, the silicone spacer was sutured and fixed, and 20 μL bacterial solution (10^8^ CFU/mL) was injected into the wound to establish an infectious full-thickness wound model. After 48 h, the wounds were treated with KC, KC@PF, and KC@PF@TA hydrogels, and were irradiated with 808 nm near infrared at 1.2 W/cm^2^ for 3 min. The wound temperature was monitored by infrared thermography before and after irradiation. The dressing was changed every two days. On days −2, 0, 3, 7, 13, and 21, the wounds were photographed to observe the healing. Statistical analysis was performed using Image J software, and the Wound healing rate was calculated according to the following formula:

(1–6) $$\text{Wound}\;\text{area}\%=A_{t}/A_{0}\times 100\%$$

where A_t_ and A_0_ represent the wound area at different time points and day −2, respectively.

### 2.14 Histological, immunohistochemical (IHC), and immunofluorescence (IF) staining measurements

After 21 days, the mice were euthanized, and the skin tissue surrounding the wound was carefully excised and immediately preserved in 4 % paraformaldehyde at 4 °C. The tissues were then embedded in paraffin and sectioned for histological examination. Hematoxylin and Eosin (HE) staining, along with Masson's trichrome staining, were performed on the tissue sections. Additionally, IHC and IF staining were conducted. CD163 (1:300, Bioss, Beijing, China) was used for IHC analysis, while α-SMA (1:200, GTX, CA, USA), CD31 (1:100, GTX, CA, USA), Rabbit anti-Mouse H&L/AF488 (1:500, Bioss, Beijing, China) and Donkey anti-Rabbit H&L/AF594 (1:500, Bioss, Beijing, China) antibodies were employed for IF staining. The stained sections were examined and imaged using an KFBIO Digital pathology slide scanner (KF-FL-020).

### 2.15 ELISA

The levels of TNF-α, IL-6, and VEGF in the skin wound tissues of 21-day post-treatment mice were quantified using ELISA kits, following the manufacturer's instructions.

### 2.16 Western blotting

Wound samples were collected from the mice on day 21 post-sacrifice and homogenized in RIPA lysis buffer containing 1 mM PSMF. The resulting protein extracts were quantified using the BCA assay. Proteins were separated via 10 % SDS-PAGE after denaturation at 95 °C for 10 min and subsequently transferred to a 0.45 μm PVDF membrane. The membrane was blocked with 5 % non-fat milk for 2 h, followed by overnight incubation at 4 °C with the primary antibodies. The membrane was then incubated with horseradish peroxidase-conjugated secondary antibodies for 2 h at room temperature. The protein bands were visualized using an electrochemiluminescence reagent and imaged with the Chemiluminescence imaging system (FUSION FX, VILBER, SWE). The antibodies used included GAPDH (1:5000, ET1601-4), CD163 (1:1000, Bioss, Beijing, China), α-SMA (1:2000, GTX, CA, USA), and HRP-labeled goat anti-mouse/rabbit IgG (1:5000, Yamei, Shanghai, China).

### 2.17 Statistics

The data were expressed as means ± standard deviation (SD). Differences between two groups were measured by Student's t-tests, and differences between multiple groups by One-way Analysis of Variance (ANOVA) followed by a Tukey post hoc test for pairwise comparison. Analyses were conducted with GraphPad Prism 9.0. p < 0.05 was considered statistical significance. (∗p < 0.05, ∗∗p < 0.01, ∗∗∗p < 0.001, ∗∗∗∗p < 0.0001).

## 3 Results and discussion

### 3.1 Synthesis and characterization of Ti₃C₂Tx-Ag composite

**Fig. 1:**
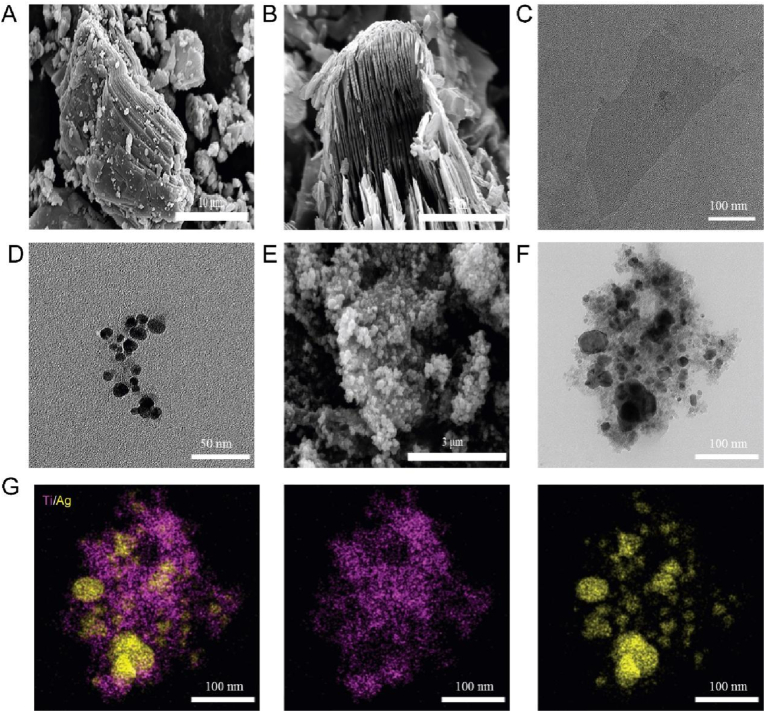
**Synthesis of the Ti₃C₂Tₓ-Ag composite.** SEM images of (A) Ti_3_AlC_2_, (B) m-Ti_3_C_2_T_x_, (E) Ti_3_C_2_T_x_-Ag. TEM images of (C) u-Ti_3_C_2_T_x_, (D) Ag NPs, (F) Ti_3_C_2_T_x_-Ag. (G) EDS mappings for Ti, Ag elements of (F).

The preparation process of Ti₃C₂Tx-Ag nanocomposites is illustrated in Fig. 1. Initially, the compact structure of Ti₃AlC₂ (Fig. 1A) was subjected to HF etching, which removed the Al layer and transformed it into a multilayered, hollow accordion-like structure of m-Ti₃C₂Tx (Fig. 1B) [45,46]. Following TMAOH intercalation and ultrasonic exfoliation, u-Ti₃C₂Tx nanosheets approximately 300 nm in size were obtained (Fig. 1C). To optimize silver loading, we determined that a u-Ti₃C₂Tx to Ag ratio of 1:10 led to supersaturation; thus, a 1:5 ratio was selected for subsequent experiments (Figure S1-2) [47]. The presence of abundant active -OH groups on the u-Ti₃C₂Tx surface may have contributed to the non-uniform size of AgNPs reduced by sodium citrate (Fig. 1D–F) [48]. Elemental analysis confirmed the successful deposition of AgNPs on the u-Ti₃C₂Tx nanosheets (Fig. 1G).

**Fig. 2:**
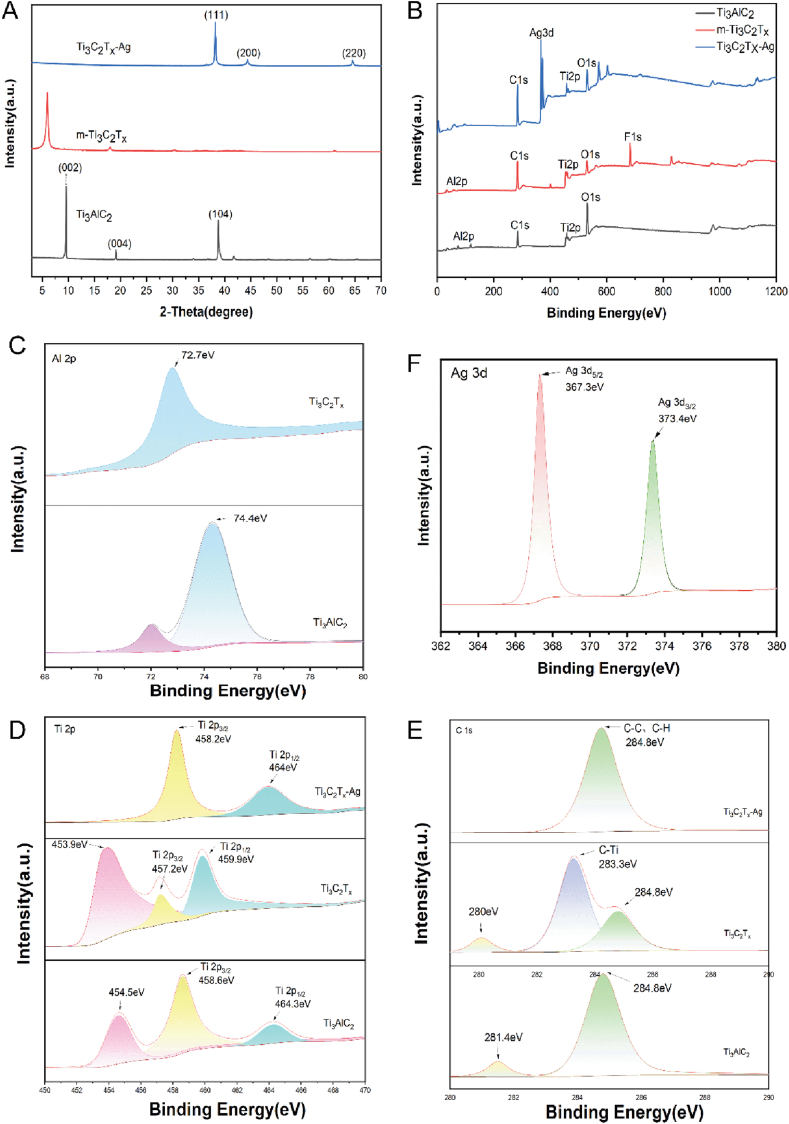
**Characterization of nanocomposite.** Ti_3_AlC_2_, m-Ti_3_C_2_T_x_, Ti_3_C_2_T_x_-Ag's (A) XRD patterns, (B) XPS spectra, and high-resolution XPS spectra of (C) Al 2p, (D) Ti 2p, (E) C 1s, (F) Ag 3d.

XRD analysis revealed significant structural changes: the (104) peak of Ti₃AlC₂ completely disappeared after HF etching, indicating the removal of the Al layer [49]. The reduction in intensity and shift of the (004) and (002) peaks suggested alterations in layer spacing and a decrease in structural order. Additionally, the appearance of (111), (200), and (220) peaks, characteristic of silver, confirmed the successful integration of AgNPs with the u-Ti₃C₂Tx nanosheets and indicated high crystallinity (Fig. 2A) [50,51]. These findings collectively demonstrate the structural transformation of Ti₃AlC₂ through various processing steps and the effective incorporation of AgNPs. Further insights were provided by XPS analysis, which showed new peaks (e.g., C 1s at 283.3 eV, 280 eV) following the removal of the Al layer, indicating changes in the bonding environment of C and Ti [52]. The leftward shift in the Ti 2p peak position suggested modifications in the chemical environment, potentially due to the formation of surface termination groups such as -OH, -F, or -Cl [53]. The slightly lower binding energies of Ag 3d (367.3 eV and 373.4 eV) compared to pure silver suggest the formation of surface chemical bonds between Ag 3d and Ti₃C₂Tx [54]. The rightward shift of the Ti 2p peak further supports this interaction (Fig. 2B–F) [55].

### 3.2 Preparation and characterization of KC@TA hydrogel

**Fig. 3:**
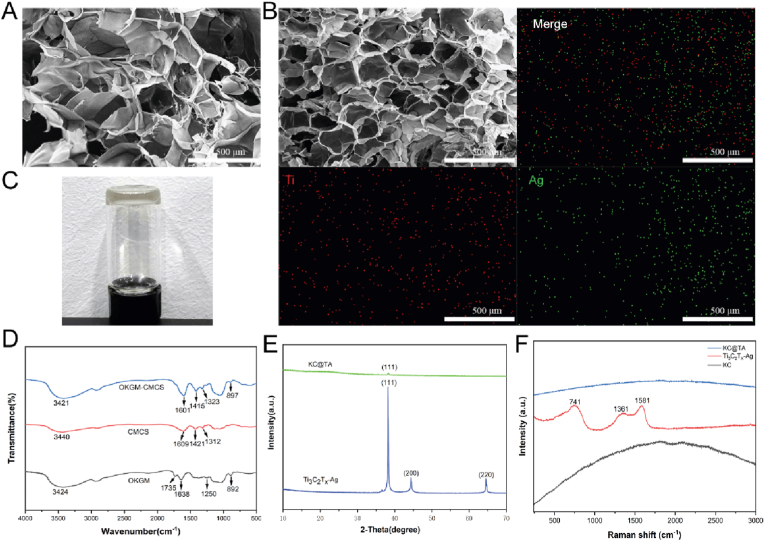
**Preparation and Characterization of hydrogels.** SEM images of (A) KC, (B) KC@TA hydrogels and EDS mappings for Ti, Ag and merge. (C) Digital photograph of the KC@TA hydrogel at room temperature. (D) FT-IR spectra of OKGM, CMCS and OKGM-CMCS. (E) XRD patterns of Ti_3_C_2_T_x_-Ag, KC@TA. (F) Raman spectra of KC、Ti_3_C_2_T_X_-Ag and KC@TA composite hydrogel materials.

The resulting KC hydrogel displayed a distinctive 3D pore structure, which (Fig. 3A). Upon introducing the Ti_3_C_2_T_x_-Ag nanocomposite, the KC@TA hydrogel presented a dark green appearance and it' s pores became significantly smaller, leading to a more compact 3D architecture (Fig. 3B). Elemental analysis verified the successful incorporation of Ti_3_C_2_T_x_-Ag, thereby constructing the KC@TA hydrogel (Fig. 3B). FT-IR spectroscopy revealed slight shifts in the O-H and N-H bonds at 3421 cm⁻^1^, likely due to hydrogen bonding changes from cross-linking [56,57]. Changes in the position and intensity of the C=O peak at 1601 cm⁻^1^ suggest the formation of new amide bonds or carboxyl groups [58,59], confirming the successful cross-linking of CMCS and OKGM and the creation of a hydrogel with a novel 3D network while retaining its original chemical characteristics (Fig. 3D). Additionally, the introduction of Ti₃C₂Tx-Ag resulted in characteristic peaks at the (111) crystal plane, confirming its successful loading into the KC hydrogel (Fig. 3E). Further structural analysis using Raman spectroscopy revealed elevated background intensity and weak characteristic peaks in the KC@TA hydrogel, suggesting a low Ti₃C₂Tx-Ag loading that masked its signals but still indicated its presence (Fig. 3F). To further verify this, XPS analysis detected that KC@TA displayed a distinct C-Ti peak at 282.9 eV, compared to the weaker 283.0 eV signal in KC, confirming the formation of C-Ti bonds and the successful integration of Ti₃C₂Tx-Ag into the KC matrix (Figure S3A). Moreover, the O 1s spectra shifted from 531.1 eV (KC) to 530.8 eV (KC@TA), indicating the formation of Ti-O/Ag-O bonds through interactions between KC oxygen groups and Ti₃C₂Tx-Ag (Figure S3B). Collectively, these results demonstrated the successful incorporation of Ti₃C₂Tx-Ag into the KC hydrogel matrix, forming stable C-Ti and Ti-O/Ag-O bonds that enhance the structural integrity of the composite hydrogel.

**Fig. 4:**
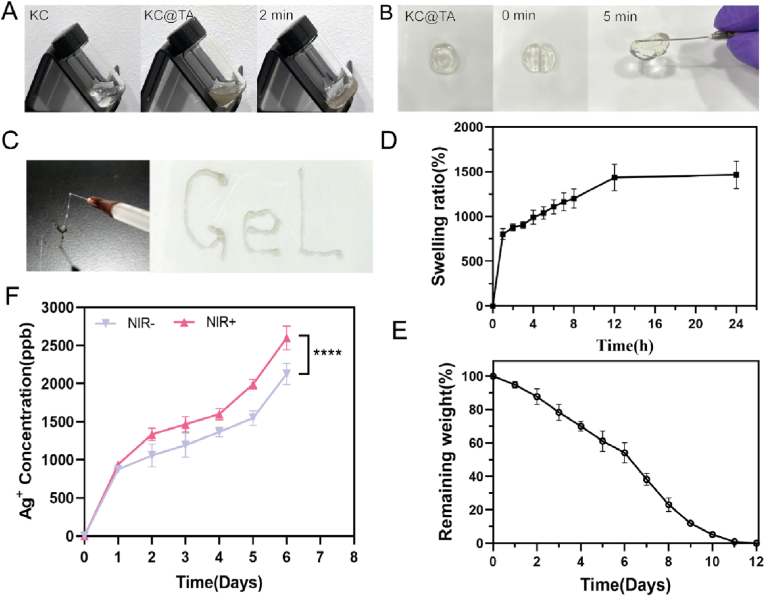
Comprehensive performance testing of KC@TA hydrogel. KC@TA hydrogel's Photographs of (A) formation process, (B) self-healing process, (C) injectable performance. Plots of (D) Swelling ratio% and (E) Remaining weight% of hydrogel as a function of time. (F) Curves of Ag^+^ concentration released over time with and without NIR irradiation (808 nm, 1.2W/cm^2^, 10 min) (∗∗∗∗p < 0.0001, n = 3).

The crosslinking of CMCS, OKGM, and Ti_3_C_2_T_x_-Ag occurred rapidly, forming hydrogels within 2 min (Fig. 4A), and these hydrogels demonstrated excellent self-healing properties, as segmented hydrogels reformed into a complete structure within 5 min at room temperature (Fig. 4B). Furthermore, the hydrogel's good injectability makes it suitable for treating irregular wounds (Fig. 4C). The KC@TA hydrogel swelled quickly within the first hour, with the swelling rate continuing to increase over 12 h, nearly reaching equilibrium at 24 h (Fig. 4D). The degradation process was slow, taking approximately 11 days for complete degradation (Fig. 4E). Additionally, Ag^+^ were continuously released from the hydrogel (Fig. 4F). These findings indicate that the KC@TA hydrogel's high water absorption and slow degradation rate promote a moist environment conducive to wound healing, while also enabling sustained drug release, reducing the need for frequent dressing changes, and enhancing therapeutic outcomes.

### 3.3 Photothermal performance of composite and hydrogel

**Fig. 5:**
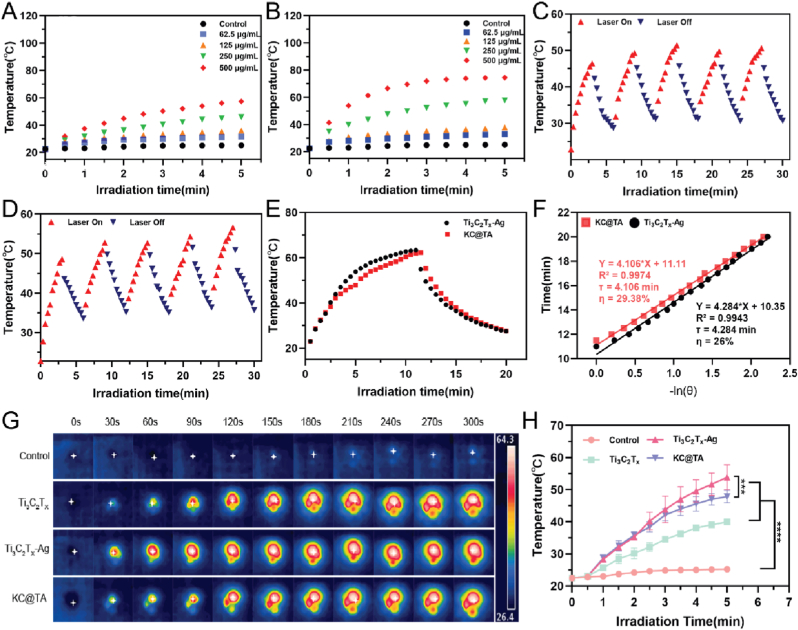
**Investigation of the photothermal properties of nanocomposites and hydrogels.** Temperature evolution curves of suspensions containing different concentrations of (A) Ti_3_C_2_T_x_, (B) Ti_3_C_2_T_x_-Ag under NIR irradiation (808 nm, 1.2 W/cm^2^, 5 min). Temperature variation of (C) Ti_3_C_2_T_x_ -Ag and (D) KC@TA hydrogel during five on/off cycles of laser irradiation. (E) Heating and cooling temperature curves of Ti_3_C_2_T_x_ -Ag and KC@TA hydrogel aqueous solution under laser irradiation. (F) Plot of cooling time versus negative natural logarithm of temperature driving force. (G) Representative infrared thermographs of H_2_O, Ti_3_C_2_T_x_, Ti_3_C_2_T_x_ -Ag, KC@TA hydrogel at different time points during NIR irradiation and (H) quantitative analysis (∗∗∗p < 0.001, ∗∗∗∗p < 0.0001, n = 3).

To evaluate the photothermal conversion capabilities of the samples, a series of experiments were conducted under varying conditions. The photothermal efficiency of the nanomaterials and their composite hydrogels was examined, revealing a concentration-dependent temperature increase for both Ti₃C₂Tx and Ti₃C₂Tx-Ag at concentrations of 62.5 μg/mL, 125 μg/mL, 250 μg/mL, and 500 μg/mL when exposed to 808 nm near-infrared light at 1.2 W/cm^2^. The control group (pure water) exhibited minimal temperature change, underscoring the significant photothermal activity of the nanocomposites (Fig. 5A and B). Cyclic laser on/off experiments demonstrated the stability and reproducibility of the photothermal effects in both Ti₃C₂Tx-Ag and KC@TA hydrogels (Fig. 5C and D). The materials consistently showed a temperature increase during laser irradiation, followed by a rapid decrease once irradiation ceased, indicating their durability through repeated photothermal cycles. Notably, KC@TA consistently achieved higher temperatures than Ti₃C₂Tx-Ag alone, suggesting that the hydrogel matrix enhances photothermal conversion by improving the dispersion and stability of the nanomaterials.

The temperature profiles of Ti₃C₂Tx-Ag and KC@TA during 20 min of irradiation (Fig. 5E) showed that KC@TA reached peak temperature more slowly, likely due to the hydrogel matrix modulating the photothermal conversion process. This results in a more gradual and stable temperature response, beneficial for photothermal applications. Subsequent analysis involved calculating the time constant (τ) and photothermal conversion efficiency (η) using linear fitting of the cooling time against the natural logarithm of the temperature difference ratio (−ln(θ)). The slope indicated a τ value of 4.106 min for KC@TA, slightly higher than the 4.284 min for Ti₃C₂Tx-Ag (Fig. 5F). Howvever, the photothermal conversion efficiency of the KC@TA hydrogel was calculated to be 29.39 % (Fig. 5F), confirming that the hydrogel contributes to enhanced photothermal performance. The significant improvement in photothermal conversion efficiency upon incorporating Ti₃C₂Tx-Ag into the KC hydrogel matrix is attributed to the synergistic interaction between the hydrogel and the nanomaterial, which likely results in improved thermal insulation and more effective photothermal energy distribution. Additionally, infrared thermography of the samples further validated that incorporating Ti₃C₂Tx-Ag into the KC hydrogel matrix effectively moderated the rapid temperature rise observed with Ti₃C₂Tx-Ag alone, providing a more controlled and uniform photothermal response (Fig. 5G and H). This indicates that the hydrogel matrix not only enhanced thermal stability but also regulated heat distribution, showcasing the distinctive synergistic effects between the hydrogel and the nanocomposite material.

### 3.4 In vitro antibacterial properties of hydrogel

To investigate the antibacterial properties of the hydrogels, three formulations were designed: the KC hydrogel, KC@T_200_ (a hydrogel incorporating 200 μg/mL Ti₃C₂Tx), and KC@TA_200_ (a hydrogel containing 200 μg/mL Ti₃C₂Tx-Ag). Ti₃C₂Tx, with its sharp edges and ability to generate ROS, exhibits inherent antibacterial properties by disrupting bacterial membranes and inhibiting bacterial growth [25]. Incorporating Ti₃C₂Tx into the hydrogel matrix creates a platform to assess these intrinsic antibacterial features. Additionally, Ti₃C₂Tx shows remarkable photothermal performance, absorbing NIR light and converting it into heat, which can further enhance its antibacterial activity. The temperature increase induced by NIR irradiation disrupts bacterial cell membranes, complementing the ROS generation from Ti₃C₂Tx for a more effective bactericidal effect [31]. To further enhance the antibacterial efficacy, Ag nanoparticles were introduced into the hydrogel. Ag is well known for its potent antibacterial properties, and incorporating Ti₃C₂Tx-Ag into the hydrogel matrix allowed for the evaluation of the synergistic effects between Ti₃C₂Tx and Ag.

**Fig. 6:**
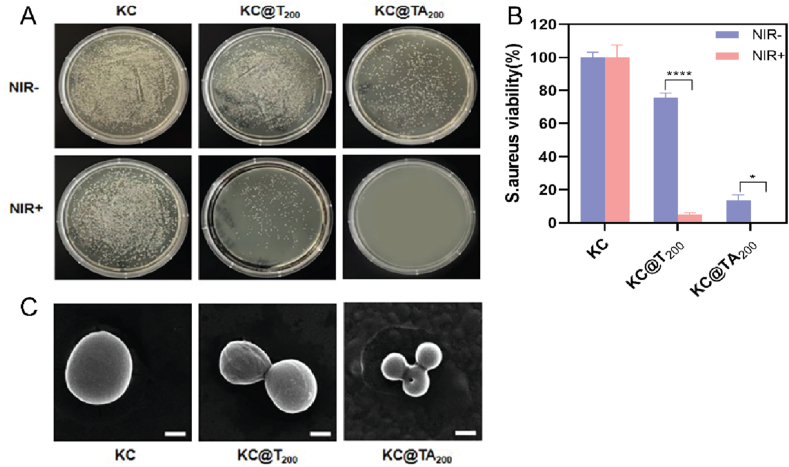
**In vitro antibacterial assay of hydrogels.** During the NIR-/NIR + irradiation (808 nm, 1.2 W/cm^2^, 15 min), (A) Photographs of *S.aureus* clones formed after co-cultured with KC, KC@T_200_ and KC@TA_200_ hydrogels for 24 h. (B) The corresponding quantitative bacterial survival viability of *S.aureus* (∗p < 0.05, ∗∗∗∗p < 0.0001, n = 3). (C) SEM images of *S.aureus* in different groups after NIR irradiation (Scale bar: 1 μm).

The antibacterial effects of KC, KC@T_200_, and KC@TA_200_ hydrogels against *S.aureus* were evaluated both with and without NIR irradiation (808 nm, 1.2 W/cm^2^). Bacterial colony formation was observed on Agar plates under both conditions. Without NIR irradiation, significant bacterial growth was noted in all samples, with a slight reduction in colonies for KC@T_200_ and the fewest colonies in the KC@TA_200_ group compared to KC. Under NIR irradiation, no bacterial colonies were observed in the KC@TA_200_ group. Statistical analysis revealed that bacterial activity was reduced to approximately 10 %, significantly lower than in the other two groups, demonstrating the superior photothermal antibacterial activity of the TA-modified hydrogel (Fig. 6A and B). Furthermore, SEM images of *S.aureus* provided additional evidence of this enhanced antibacterial effect. In the KC group, the bacteria maintained a smooth, spherical morphology after NIR irradiation. In contrast, bacteria treated with KC@T_200_ exhibited partial deformation, while those treated with KC@TA_200_ showed severe deformation and destruction, further confirming the improved antibacterial performance of the KC@TA_200_ hydrogel (Fig. 6C).

### 3.5 PF promoted HUVECs proliferation and migration

In the design of biomaterials aimed at promoting diabetic wound healing, it's important to address more than just antibacterial properties. While the KC@TA hydrogel exhibits excellent photothermal antibacterial activity, effective wound repair also requires enhanced cell proliferation, migration, and tissue regeneration—factors that KC@TA alone may not fully provide.

**Fig. 7:**
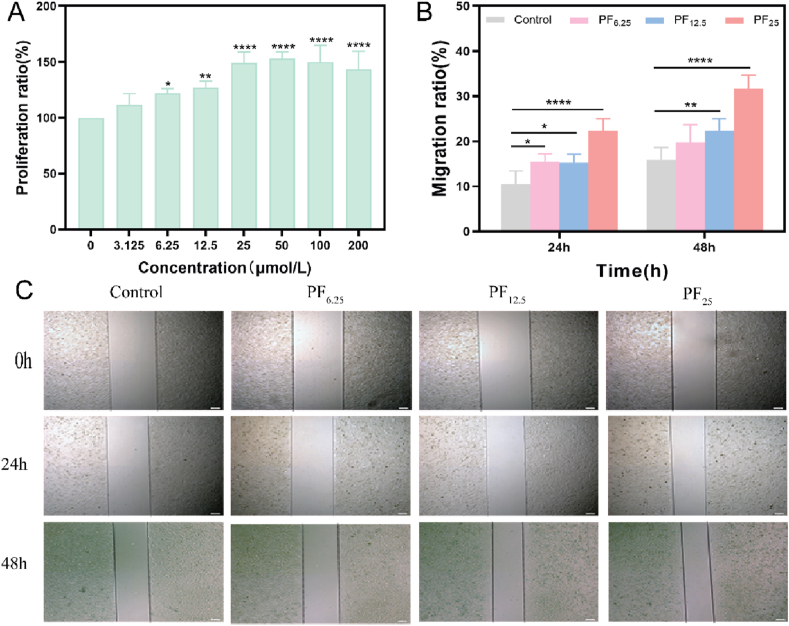
**Evaluation of proliferation and migration of HUVECs treated with different concentrations of PF *in vitro*.** (A) Proliferation ratio results of HUVECs with different concentrations of PF. (B) The migration ratio of HUVECs treated with PF and (C) the representative images after 0 h, 24 h, and 48 h (∗p < 0.05, ∗∗p < 0.01, ∗∗∗∗p < 0.0001, n = 4; Scale bar: 200 μm).

PF, an active compound extracted from Paeonia, is known for its broad biological activities, particularly its anti-inflammatory, antioxidant, and cell proliferation-promoting effects [60,61]. To further investigate its potential, we examined the impact of varying concentrations of PF on cell proliferation and migration. Experimental data revealed that HUVECs treated with PF at concentrations ranging from 3.125 to 200 μmol/L for 48 h exhibited a dose-dependent increase in proliferation, with a significant rise starting at 12.5 μmol/L and peaking at 200 μmol/L (Fig. 7A). Additionally, we assessed cell migration at low (6.25 μmol/L), medium (12.5 μmol/L), and high (25 μmol/L) PF concentrations over 0, 24, and 48 h. The results indicated a marked increase in migration rates over time, particularly at 48 h, with the highest rate observed at 25 μmol/L PF (Fig. 7B and C). These findings suggest that PF not only promotes cell proliferation but also accelerates cell migration, both of which are crucial for effective wound healing.

Given these benefits, we developed the KC@PF@TA hydrogel, integrating the cell-proliferative properties of PF with the photothermal antibacterial activity of KC@TA, for further experimental evaluation.

### 3.6 In vitro biocompatibility of hydrogel

**Fig. 8:**
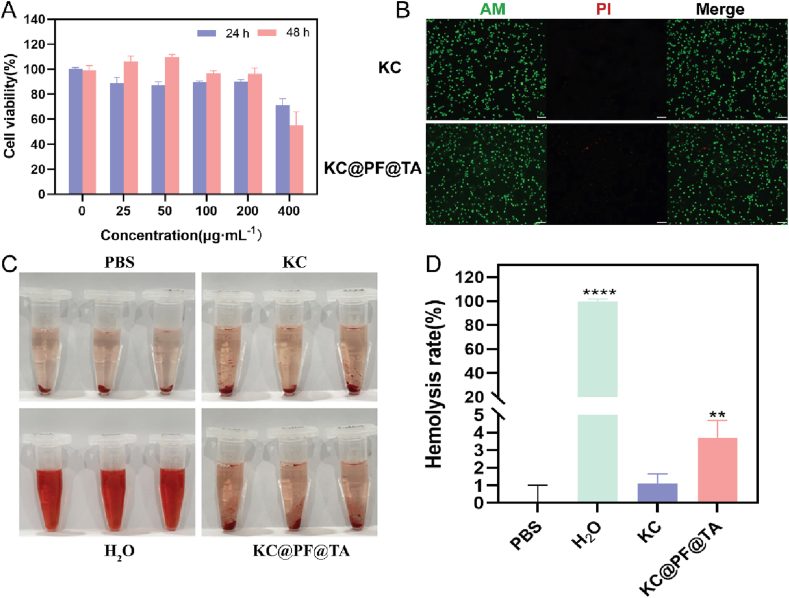
***In vitro* biocompatibility examination and hemolytic test of NIH3T3 under hydrogels.** (A) Cells toxicity results of NIH3T3 treated with different concentrations of Ti_3_C_2_T_X_-Ag in KC@PF@TA hydrogels after 24 h and 48 h. (B) Representative images of Calcein-AM/PI-stained cells treated with 200 μg/mL hydrogel for 48 h (Scale bar: 100 μm). (C) Representative pictures of hemolysis and (D) quantitative analysis results of hemolysis rate of hydrogels after contact with RBCs for 30min (∗∗p < 0.01, ∗∗∗∗p < 0.0001, n = 3).

To ensure the safety of hydrogels in biomedical applications, it is crucial to evaluate their biosafety, specifically regarding potential cytotoxicity or other adverse reactions when used in vivo. This assessment guarantees their effectiveness and safety as wound repair materials. We examined the cytotoxicity and hemolysis of KC@PF@TA hydrogels with varying concentrations of nanomaterials (0, 25, 50, 100, 200, 400 μg/mL) (Fig. 8). The results revealed that, except for the 400 μg/mL group, all other groups maintained over 75 % cell viability after 24 h of co-culture, indicating good cytocompatibility. However, at 48 h, cell viability in the 400 μg/mL group dropped to 54.86 %, highlighting significant cytotoxicity at this concentration (Fig. 8A). Consequently, 200 μg/mL was selected as the optimal nanomaterial concentration, balancing cytocompatibility and antibacterial efficacy. Further biocompatibility testing at 200 μg/mL using Calcein-AM/PI staining demonstrated that only a few dead cells were observed after 48 h of co-culture, suggesting minimal cytotoxic impact at this concentration (Fig. 8B). Such excellent biocompatibility is critical for ensuring the safety of the hydrogel in practical applications. Moreover, the hemolysis rate of KC@PF@TA hydrogel was approximately 3.17 %, significantly below the 5 % threshold required for biomedical materials (Fig. 8C and D) [62]. This low hemolysis rate indicates that the hydrogel has minimal adverse effects on red blood cells, further supporting its safety for in vivo use.

In summary, the KC@PF@TA hydrogel demonstrated strong biocompatibility and low hemolysis at appropriate nanomaterial concentrations, confirming its potential as a safe and effective material for wound repair.

### 3.7 Diabetic infected wound healing performance of KC@PF@TA hydrogel in vivo

In vitro studies indicate that the KC@PF@TA hydrogel shows significant potential for repairing diabetic wounds. To further explore this, C57BL/6J mice were administered STZ daily for five consecutive days, leading to stable blood glucose levels >16.7 mM and the successful establishment of a diabetic mouse model [63]. Following this, a 6 mm full-thickness wound was created on the backs of the mice, and then infected with *S.aureus* 48 h before treatment with the hydrogels. Previous studies have shown that there is no significant difference between KC hydrogel and blank control treatment in infected wounds [64]. Therefore, the wounds were treated with KC, KC@PF, and KC@PF@TA hydrogels.

**Fig. 9:**
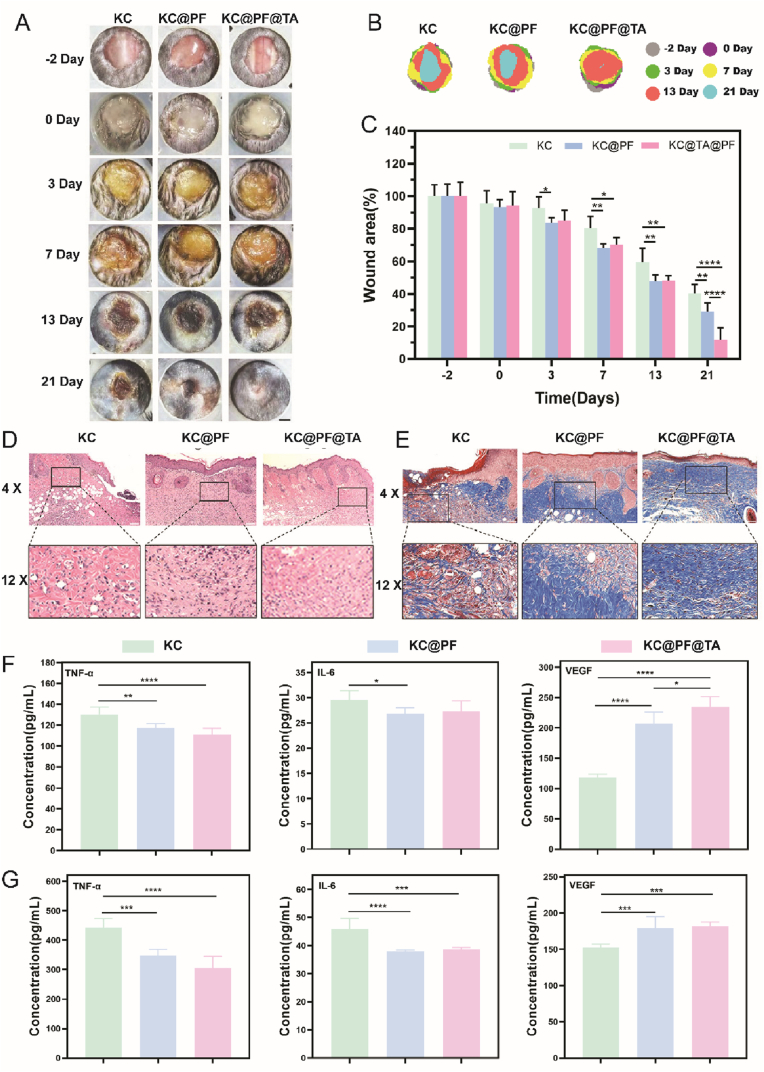
**Effects of hydrogels in promoting diabetic wound healing in C57Bl/6J mices.** (A) Representative images of the wound at day -2, 0, 3, 7, 13, 21 and (B) the diagrams of time-evolved wound areas (Scale bar: 20 mm). (C) Statistical analysis of the wound area (∗p < 0.05, ∗∗p < 0.01, ∗∗∗∗p < 0.0001, n = 6). (D) HE staining and (E) Masson's trichrome staining of wound tissues on day 21. Results of ELISA for the expression levels of TNF-α, IL-6, and VEGF in (F) serum and (G) wound tissues (∗p < 0.05, ∗∗p < 0.01, ∗∗∗p < 0.001, ∗∗∗∗p < 0.0001, n = 4).

As illustrated in Fig. 9A and B, the wounds treated with KC@PF and KC@PF@TA hydrogels exhibited significantly faster healing rates compared to the blank hydrogel (KC group). Initially, there was minimal difference in healing rates between the KC@PF and KC@PF@TA groups until day 13. However, from day 13 to day 21, the wounds treated with KC@PF@TA hydrogel demonstrated accelerated healing, reaching a healing rate of approximately 88.12 % by day 21 (Fig. 9C). This finding suggests that, while there was no significant difference in healing rates between the KC@PF and KC@PF@TA groups compared to the blank hydrogel, the drug-loaded hydrogel effectively shortened the inflammatory phase, facilitating the transition to the proliferative phase of healing. Importantly, the KC@PF@TA hydrogel also enhanced the tissue remodeling phase.

Histological analysis through HE staining corroborated the healing rates, with the KC@PF@TA group displaying the best healing status on day 21, characterized by increased epidermal thickness and reduced wound length (Fig. 9D). The deposition of collagen, both in quality and quantity, plays a crucial role in determining the speed and efficacy of wound healing, making it a key indicator of healing progress [65]. The formation of new collagen was visualized using Masson's trichrome staining. On day 21, remnants of scabs were still present in the KC group, with an incomplete epidermal structure. In the KC@PF group, the epidermis appeared clearer and more complete; however, it was thicker, with heterogeneous and disorganized collagen fibers. In contrast, wounds treated with the KC@PF@TA hydrogel exhibited an intact epidermis resembling healthy skin, along with abundant new collagen and uniformly arranged collagen fibers (Fig. 9E). This suggests that the KC@PF@TA hydrogel-treated wounds closely resemble healthy skin on day 21, demonstrating effective healing and an accelerated remodeling phase.

In diabetic wounds, excessive inflammation and disordered angiogenesis significantly hinder the healing process. Therefore, we evaluated the impact of the KC@PF@TA hydrogel on inflammatory factors (IL-6, TNF-α) and growth factors (VEGF). As shown in Fig. 9F and G, the treatment with KC@PF and KC@PF@TA hydrogel resulted in a significant reduction in the expression levels of IL-6 and TNF-α in both serum and wound tissues, while VEGF expression was notably increased. These findings indicate that both hydrogels effectively inhibited inflammatory responses and promoted neovascularization and tissue regeneration.

### 3.8 KC@PF@TA hydrogel promoted wound healing by anti-inflammation and pro-angiogenesis

**Fig. 10:**
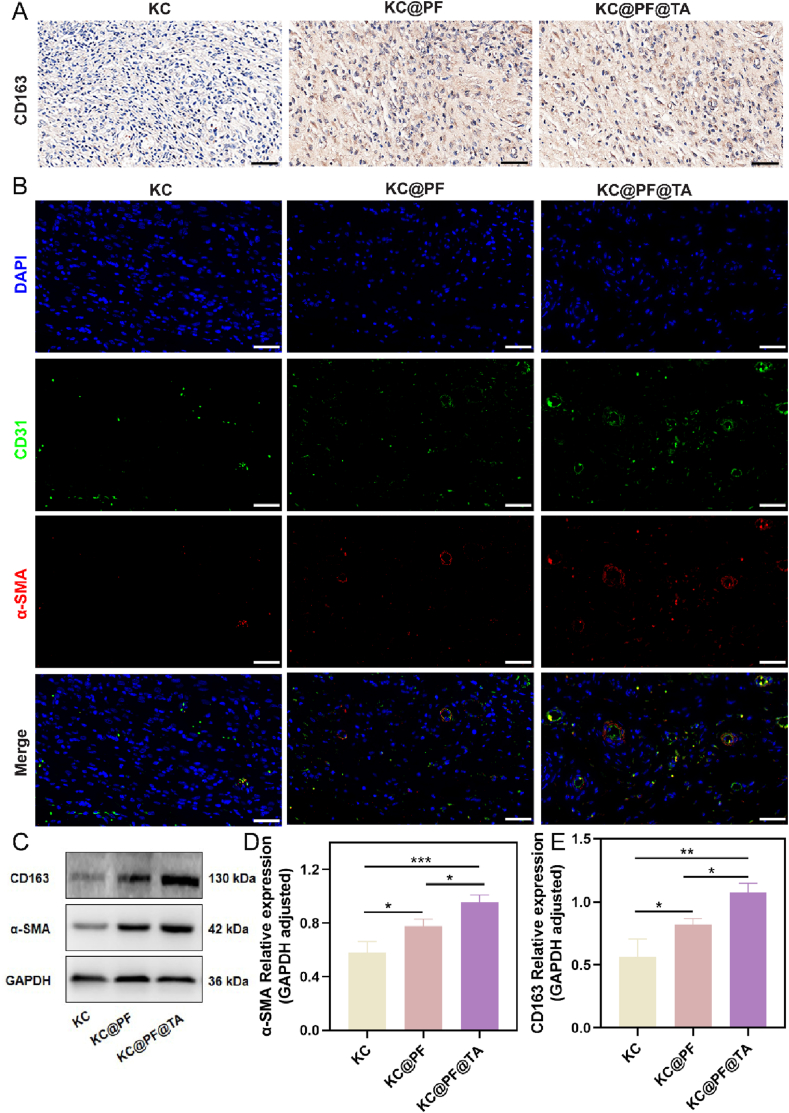
**KC@PF@TA hydrogel enhanced M2 macrophage activity and promoted angiogenesis.** (A) Immunohistochemical staining of CD163 in regenerated skin tissue on day 21 (Scale bar: 200 μm). (B) Immunofluorescence staining results of DAPI (blue), CD31 (green), *α*-SMA (red) and merge on day 21 (Scale bar: 50 μm). (C) Western blot analysis of GAPDH, α-SMA, CD163, and the quantitative analysis of (D) α-SMA, (E) CD163 (∗p < 0.05, ∗∗p < 0.01, ∗∗∗p < 0.001, n = 3).

Subsequently, we investigated the role of immune modulation mediated by the KC@PF@TA hydrogel in facilitating wound healing. M2 macrophages, known as "repair" macrophages, play a critical role in tissue repair and anti-inflammatory responses. To assess the involvement of M2 macrophages in wound healing, we examined the expression of the M2 macrophage marker CD163. As shown in Fig. 10A, the KC group exhibited lower CD163 expression, suggesting weaker M2 macrophage activity, which may have contributed to delayed wound healing due to inadequate immune regulation. In contrast, the KC@PF group demonstrated a significant increase in CD163 expression, indicating that the addition of PF effectively activated M2 macrophages, enhancing their immunomodulatory function and thereby accelerating wound repair. Notably, the KC@PA@TA group showed even higher CD163 expression, highlighting the synergistic effect of PF and Ti_3_C_2_T_x_-Ag in promoting M2 macrophage activity. This enhanced immunomodulation likely contributed to faster tissue repair and wound healing. Overall, the qualitative analysis revealed that incorporating PF and Ti_3_C_2_T_x_-Ag into the hydrogel significantly boosted M2 macrophage activity, leading to more efficient wound healing. To substantiate the qualitative IHC findings, we conducted a quantitative analysis of CD163 protein expression using WB. The results were consistent with the IHC observations, further confirming the pivotal role of M2 macrophages in wound healing through immune regulation (Fig. 10C–E).

During the proliferative phase of wound healing, blood vessels are essential for supplying nutrients and oxygen to cells, thereby facilitating wound repair [66]. To assess angiogenesis, we performed immunofluorescence analysis on day 21. As shown in Fig. 10B, the KC@PF@TA hydrogel-treated wounds exhibited more mature blood vessels, with longer vessels and larger lumens, indicating that this hydrogel promotes angiogenesis. Additionally, WB results demonstrated an increase in α-SMA expression in wounds treated with KC@PF and KC@PF@TA (Fig. 10C and D), signifying the activation of myofibroblasts. Myofibroblasts are crucial for wound healing and remodeling due to their ability to synthesize extracellular matrix and contract wound tissue. The observed elevation in α-SMA expression may also be linked to increased collagen synthesis, further supporting tissue remodeling and trauma healing [67].

## 4 Conclusion and discussion

In this study, a novel KC@PF@TA nanocomposite hydrogel was developed, demonstrating significant potential for diabetic wound repair. The integration of Ti₃C₂Tx-Ag into the hydrogel enhanced its photothermal conversion efficiency and antibacterial properties, while PF supported cell proliferation and migration with excellent biocompatibility. Both in vitro and in vivo studies confirmed the hydrogel's ability to modulate inflammation, promote angiogenesis, and facilitate tissue remodeling, all of which contributed to the accelerated wound healing. Notably, PF played a key role in supporting these processes, enhancing the overall therapeutic efficacy of the hydrogel. These results highlight the KC@PF@TA hydrogel as a promising and safe candidate for clinical wound care applications.

Despite the promising results, several challenges and areas for improvement remain. The hydrogel's efficacy and safety have only been evaluated in vitro and in mouse models; its performance in larger animal models or clinical trials needs further validation. Additionally, while the hydrogel shows good biocompatibility and degradability under experimental conditions, its long-term stability and the effects of its degradation products on the body require further investigation. Future research should focus on optimizing the hydrogel's composition and preparation process to enhance mechanical strength and controlled release, addressing the needs of various diabetic wound types. What's more, incorporating active ingredients with anti-inflammatory, antioxidant, and pro-angiogenic properties could further enhance its therapeutic efficacy. Exploring the intelligent responsiveness of multifunctional hydrogels to stimuli such as temperature and pH presents exciting opportunities for developing advanced, personalized wound care materials.

## CRediT authorship contribution statement

**Xue Ou:** Writing – review & editing, Writing – original draft, Visualization, Methodology, Conceptualization. **Zhijie Yu:** Writing – review & editing, Supervision, Conceptualization. **Xi Zheng:** Validation, Resources. **Le Chen:** Methodology, Conceptualization. **Chuanyu Pan:** Validation, Methodology. **Dandan Li:** Investigation, Formal analysis, Conceptualization. **Zhenzhen Qiao:** Software, Formal analysis. **Xiaoyuan Zheng:** Writing – original draft, Supervision, Resources, Project administration, Conceptualization.

## Declaration of competing interest

The authors declare that they have no known competing financial interests or personal relationships that could have appeared to influence the work reported in this paper.

## Data Availability

The data that has been used is confidential.

## Abbreviations

Ti_3_AlC_2_: aluminum carbon titanide
HF: hydrofluoric acid
TMAOH: tetramethylammonium hydroxide
AgNO_3_: silver nitrate concentrate
SC: sodium citrate
CMCs: carboxymethyl chitosan
OKGM: oxidized konjac glucomannan
TNF-α: tumor necrosis factor-α
IL-6: interleukin-6
VEGF: endothelial growth factor
SEM: scanning electron microscopy
EDS: energy dispersive spectroscopy
TEM: transmission electron microscopy
XRD: X-ray diffraction
XPS: X-ray photoelectron spectroscopy
FTIR: fourier transform infrared spectroscopy
NIR: near-infrared
ICP-MS: inductively coupled plasma mass spectrometry
PBS: phosphate buffered saline
HUVECs: human umbilical vein endothelial cells
OD: optical density
DMEM: dulbecco's modified eagle medium
STZ: streptozocin
IHC: immunohistochemical
IF: immunofluorescence
HE: hematoxylin and eosin
